# Music-evoked emotions classification using vision transformer in EEG signals

**DOI:** 10.3389/fpsyg.2024.1275142

**Published:** 2024-04-04

**Authors:** Dong Wang, Jian Lian, Hebin Cheng, Yanan Zhou

**Affiliations:** ^1^School of Information Science and Electrical Engineering, Shandong Jiaotong University, Jinan, China; ^2^School of Intelligence Engineering, Shandong Management University, Jinan, China; ^3^School of Arts, Beijing Foreign Studies University, Beijing, China

**Keywords:** music-evoked emotion, emotion classification, electroencephalographic, deep learning, transformer

## Abstract

**Introduction:**

The field of electroencephalogram (EEG)-based emotion identification has received significant attention and has been widely utilized in both human-computer interaction and therapeutic settings. The process of manually analyzing electroencephalogram signals is characterized by a significant investment of time and work. While machine learning methods have shown promising results in classifying emotions based on EEG data, the task of extracting distinct characteristics from these signals still poses a considerable difficulty.

**Methods:**

In this study, we provide a unique deep learning model that incorporates an attention mechanism to effectively extract spatial and temporal information from emotion EEG recordings. The purpose of this model is to address the existing gap in the field. The implementation of emotion EEG classification involves the utilization of a global average pooling layer and a fully linked layer, which are employed to leverage the discernible characteristics. In order to assess the effectiveness of the suggested methodology, we initially gathered a dataset of EEG recordings related to music-induced emotions.

**Experiments:**

Subsequently, we ran comparative tests between the state-of-the-art algorithms and the method given in this study, utilizing this proprietary dataset. Furthermore, a publicly accessible dataset was included in the subsequent comparative trials.

**Discussion:**

The experimental findings provide evidence that the suggested methodology outperforms existing approaches in the categorization of emotion EEG signals, both in binary (positive and negative) and ternary (positive, negative, and neutral) scenarios.

## 1 Introduction

Emotion is intricately intertwined with all facets of the human experience and action. According to Jerritta et al. (2011), it has an impact on human attitudes and perceptions in both human-human contact and human-computer interaction. In the realm of artistic expression, music holds a paramount position as a means to convey and articulate human emotions. Music has been widely recognized as a means of evoking distinct emotive states, leading to its characterization as the language of emotions (Vuilleumier and Trost, 2015). In their investigations, Ekman (1999) and Gilda et al. (2017) introduced six distinct and quantifiable emotional states, namely happiness, sadness, anger, fear, surprise, and disgust, as the basis for implementing emotion identification. Over time, other emotional states have been included in this collection, such as neutrality, arousal, and relaxation (Bong et al., 2012; Selvaraj et al., 2013; Goshvarpour et al., 2017; Minhad et al., 2017; Wei et al., 2018; Sheykhivand et al., 2020; Liu et al., 2022). In the context of machine learning, the establishment of distinct states for emotions serves as a significant framework for effectively addressing the challenge of emotion recognition. Numerous algorithms for music emotion identification based on machine learning have been proposed in the literature, with applications spanning composition and psychotherapy (Eerola and Vuoskoski, 2012; Cui et al., 2022).

Typically, a conventional music emotion identification system based on machine learning encompasses the subsequent stages:

- The collection of changes in emotions elicited by music is facilitated via the utilization of physiological information obtained by specialized sensors.
- The physiological samples that have been gathered are subjected to a processing procedure in order to remove any potential artifacts.
- The generation of representation pertaining to emotional states is thereafter accomplished by extracting features from the pre-processed data.
- By utilizing a classifier, it is possible to generate the corresponding category of music emotion for a given sample.

Numerous instruments utilized in the acquisition of physiological signals have been employed for the purpose of emotion recognition. Various physiological signals have been investigated for the purpose of emotion recognition. These include, body movement (Zhang et al., 2021), facial expression (Song, 2021), respiration (Siddiqui et al., 2021), galvanic skin response (Kipli et al., 2022), blood volume pulse (Semerci et al., 2022), skin temperature (Semerci et al., 2022), electromyography (Xu et al., 2023), photoplethysmographic (Cosoli et al., 2021), electrocardiogram (Hasnul et al., 2021), and EEG (Li et al., 2021). The non-invasive nature, affordability, and ability to capture data in real-time have contributed to the extensive utilization of EEG in the field of emotion identification (Alarcao and Fonseca, 2017), with a particular emphasis on music emotion categorization (Lin et al., 2006).

Several studies have introduced different approaches for emotion categorization utilizing EEG in the context of machine learning. For example, the study conducted by Sammler et al. (2007) examined the impact of valence on human emotions by analyzing EEG data and heart rate concurrently. The present study aimed to gather data on positive and negative emotions elicited by EEG signals during the auditory experience of consonant and discordant musical stimuli. Subsequently, the authors of the study (Koelstra et al., 2011) made available a publicly accessible dataset. The study conducted by Balasubramanian et al. (2018) examined the emotional reaction to various types of music using EEG data. The experimental findings have indicated that there is an increase in theta band activity in the frontal midline region when individuals are exposed to their preferred music. Conversely, the beta band would have an increase in activity when exposed to music that is perceived as undesirable. In their study, Ozel et al. (2019) introduced a methodology for emotion identification that involves the analysis of temporal-spectral EEG signals. Hou and Chen (2019) derived a set of 27-dimensional EEG characteristics to represent music-induced emotions, including calmness, pleasure, sadness, and rage. *Recently, Qiu et al. (2022) proposed an integrated framework of multi-modal EEG and functional near infrared spectroscopy to explore the influence of music on brain activity*.

In addition, the utilization of deep learning-based architectures in music emotion categorization has been widely adopted due to the shown effectiveness of deep learning in different domains such as machine vision and natural language processing. In their study, Han et al. (2022) conducted a comprehensive review of the existing literature pertaining to the assessment metrics, algorithms, datasets, and extracted features utilized in the analysis of EEG signals in the context of music emotion detection. In their publication, Nag et al. (2022) introduced the JUMusEmoDB dataset. The music emotion categorization challenge was addressed by the authors through the utilization of Convolutional Neural Network (CNN) based models, namely resnet50, mobilenet, squeezenet, and their own suggested ODE-Net. Eskine (2022) conducted a study examining the impact of music listening on creative cognition, a phenomenon that has been empirically demonstrated to enhance creative cognitive processes. The experimental findings provided evidence that cognitive function exhibited an increase inside the default mode. This was supported by the observed augmentation of spectral frequency power in the beta range throughout the entire brain, as well as in the theta range within the parietal region, and in the gamma range across the entire brain. In their study, Daly (2023) investigated the integration of functional magnetic resonance imaging (fMRI) and EEG techniques to develop an acoustic decoder for the purpose of classifying music emotions. The study employed an EEG-fMRI combined paradigm to capture neural responses during music listening among individuals. In this study, a deep learning model known as the long short-term memory (LSTM) was utilized to extract neural information from EEG signals during music listening. The objective was to rebuild the matching music clips based on this extracted information.

Both machine learning and deep learning techniques have demonstrated promising results in the categorization of music-evoked emotions. Nevertheless, there are a number of constraints associated with these approaches that must be addressed prior to their practical implementation in contexts such as medical diagnosis, namely in the realm of emotion identification. One aspect to consider is that the efficacy of machine learning techniques is heavily dependent on the selection of appropriate features. The task at hand continues to provide an unsolved problem as the extraction and selection of these characteristics from EEG data must be done in a manual manner. In addition, it should be noted that manually-designed features possess subjectivity and susceptibility to errors, perhaps rendering them unsuitable for the specific requirements of music emotion identification. In contrast, deep learning models like as CNNs have the ability to automatically extract internal representations from EEG inputs. Nevertheless, it is expected that the features derived from CNN models prioritize the consideration of the overall connection between distant EEG signals. This is due to the fact that CNN utilizes a local receptive field approach in the process of extracting features.

The present work introduces a transformer architecture for music-evoked emotion categorization, using a self-attention mechanism. This model incorporates the self-attention mechanism and positional embedding to describe the sequence of channels in EEG data, drawing inspiration from the vision transformer's work (Dosovitskiy et al., 2020). The suggested transformer model has the ability to extract both spatial representations, which correspond to self-attention modules, and temporal representations, which correspond to positional embedding. These representations are derived from multi-channel EEG data acquired from subjects who were listening to music. Furthermore, the transformer model that has been introduced has the capability to extract the relationships that exist among EEG signals across extended distances. In order to assess the efficacy of the suggested methodology, the experiments were conducted using both a publicly accessible dataset (Koelstra et al., 2011) and a privately held dataset. Furthermore, comparative tests were conducted to evaluate the performance of the proposed model in comparison to state-of-the-art algorithms. The experimental findings provide evidence that the suggested methodology exhibits superior performance compared to existing binary and ternary music emotion categorization algorithms. The suggested model has a positive conclusion, indicating its potential value as a tool for classifying music-evoked emotions.

The main contributions of this work can be summarized as follows:

- *This is an early application of the spatial-temporal transformer into the classification of music-evoked emotions*.
- *A novel dataset of music-evoked EEG signals was established*.
- *The proposed approach considers both the spatial connections among a set of EEG channels and the temporal sequence of each individual EEG signal*.
- *The performance of our approach surpassed the state-of-the-art deep learning algorithms on both public and private datasets*.

The subsequent sections of this article are structured as follows: The methodology Section 2 contains information on the acquisition of EEG signals during music listening as well as the details of the presented deep learning model. Section 3 presents a detailed account of the experimental procedures conducted in this investigation, as well as a comprehensive comparison between the existing state-of-the-art methods and the technique proposed in the current study. This research concluded at Section 4.

## 2 Methodology

This section provides a comprehensive overview of the data gathering process employed in the present investigation. Furthermore, the subsequent sections of the article will present a comprehensive analysis of the suggested transformer model.

### 2.1 Dataset and pre-processing

**Figure 1:**
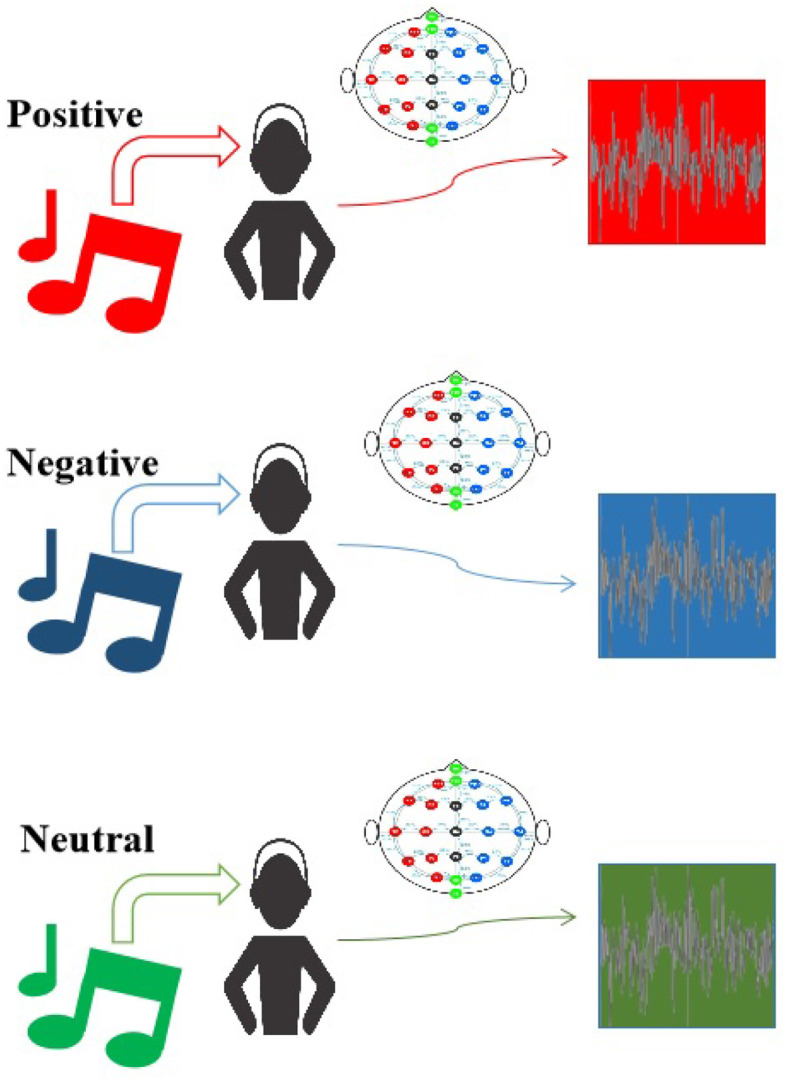
The data collecting process for classifying music-evoked emotions using an EEG equipment based on the 10–20 system (Homan et al., 1987).

The initial step in this study included the creation of a private dataset using multi-channel EEG, which involved the collection of three distinct music-evoked emotions: positive, negative, and neutral. The complete workflow is depicted in Figure 1.

During the course of data gathering, a total of 48 individuals were registered, including 24 females and 24 men. The age range of the participants was between 18 and 25 years, with an average age of 20.6. All individuals involved in the study were enrolled as students at the same institution's campus. Furthermore, it should be noted that the individuals exhibit robust physical and mental well-being. During the course of the project, the research team received advice and supervision from two psychology specialists, one female and one male, who possessed significant expertise in the field.

To ensure the consistency of the data gathering process, the following challenges were proactively addressed. Additionally, all participants were provided with instructions to thoroughly review the handbook and become acquainted with the workflow of EEG signal collecting. It should be noted that the manual has identified and emphasized the entries that are prone to errors, with the intention of facilitating the reader's attention toward the vital operations. Subsequently, the participants were requested to complete a questionnaire pertaining to their personal details. Subsequently, the participants were provided with instructions and guidance from the specialists in order to properly don the EEG electrode caps. Subsequently, the specialists would assess the adequacy of the EEG electrodes' contact and ensure that no detachment has occurred. Furthermore, the participants were instructed by the experts to initiate the signal gathering procedure by hitting the designated buttons. In addition, the EEG collection device utilized in the study was the Biosemi ActiveTwo system. The system employs the international 10–20 system, consisting of 32 channels, notably Fp1, AF3, F3, F7, FC5, FC1, C3, T7, CP5, CP1, P3, P7, PO3, O1, Oz, Pz, Fp2, AF4, Fz, F4, F8, FC6, FC, Cz, C4, T8, Cp6, Cp2, P4, P8, PO4, and O2. Additionally, the sampling rate is set at 512Hz.

During the process of data collection, each participant was provided with instructions to listen to a total of 15 music clips. These clips were categorized into three distinct emotional categories, namely positive, negative, and neutral, with each category consisting of five clips. To note that the categories of these clips were determined by three psychological experts using a majority voting mechanism. The specifics about the music may be found in Table 1. The initial duration of the music clips varies among them. Nevertheless, the participant received a standardized 1-min audio clip for each piece of music. Each participant was instructed to listen to the music clips in a randomized sequence.

**Table 1:** The descriptions of the music excerpts used in this study.

**ID**	**Type**	**Title**	**Singer**	**Durating (mm:ss)**
1	Positive	Honey	Xinling Wang	03:33
2	Negative	Advanced animals	Wei Dou	04:38
3	Neutral	Reiki meditation	Reiki	06:03
4	Positive	Wu Ha	Weibo Pan	03:46
5	Negative	In case	SHIN	04:24
6	Neutral	Calm dreams	Sleep Tech	04:27
7	Positive	In Spring	Feng Wang	05:10
8	Negative	Cloudy day	Wenwei Mo	04:02
9	Neutral	Let the sun shine	Milk & Sugar	07:02
10	Positive	As broad as the sea and sky	Beyond	03:59
11	Negative	Negative	Black sun empire	05:44
12	Neutral	Illusionary daytime	Shirfine	04:10
13	Positive	Invisible wings	Shaohan Zhang	03:44
14	Negative	Unfortunately, its not you	Jingru Liang	04:45
15	Neutral	Song from a secret garden	Secret garden	03:33

The subsequent section presents a comprehensive overview of the data collecting procedure involved in capturing EEG signals related to music-induced emotions.

(1) The participants were provided with instructions to achieve a state of calmness, following which the experts started the marking process to denote the commencement of each EEG recording. The duration of this process is expected to be 5 seconds.
(2) In each 75-second interval, the participants would undergo a 15-second pause to transition between music clips, followed by a 60-second period of actively listening to the music clip. Simultaneously, the experts would provide guidance to the participants on how to minimize superfluous bodily motions.
(3) Following the auditory experience, the individuals were directed by the experimental personnel to assign a value to the musical composition, with positive being denoted as +1, negative as –1, and neutral as 0. The duration of this procedure should not exceed 15 seconds, during which it is utilized for the purpose of transitioning the music.
(4) The participants proceeded with the auditory experience by sequentially engaging with the subsequent musical excerpt until the entirety of the 12 excerpts had been presented.

So as to guarantee the optimal state of the participants, the collection of music-evoked emotion EEG samples was limited to the time periods of 9 a.m. to 11 a.m. and 3 p.m. to 5 p.m. In order to mitigate interference from many sources such as heart rate, breathing, electrocardiogram (ECG), and electro-scalogram (EOG), the participants were given instructions to cover their eyes while the recording procedures were being conducted.

The dataset contains a total of 43,200 (48 × 15 × 60 = 43, 200) seconds of EEG signals, with each second including 32 channels. Furthermore, the initial samples were partitioned into the epochs of 1 second duration, each consisting of 60, 000 data points. To note that there were still overlapping epochs in the samples since the trivial errors are difficult to avoid due to the human reaction times. Given the absence of any imbalance issue within the dataset, it can be observed that each category of music emotion EEG signals is comprised of an equal number of samples, specifically 20,000 epochs. Hence, in the context of binary classification, namely distinguishing between positive and negative classes, the proposed model was trained using a dataset including 40,000 epochs as input samples. In contrast, in the context of the ternary classification job, the entirety of the 60,000 epochs were utilized as the input. It should be noted that the presence of overlapping epochs has the potential to somewhat mitigate over-fitting.

In the pre-processing phase, the acquired EEG signals were subjected to a Notch filter (Serra et al., 2017) in order to remove the 50 Hz components originating from the power supply. Subsequently, a first-order low-pass filter with a frequency range of 0.5 to 45 Hz was utilized. Subsequently, the electroencephalography (EEG) data underwent a normalization process resulting in a range of values between 0 and 1.

### 2.2 The proposed transformer architecture

**Figure 2:**
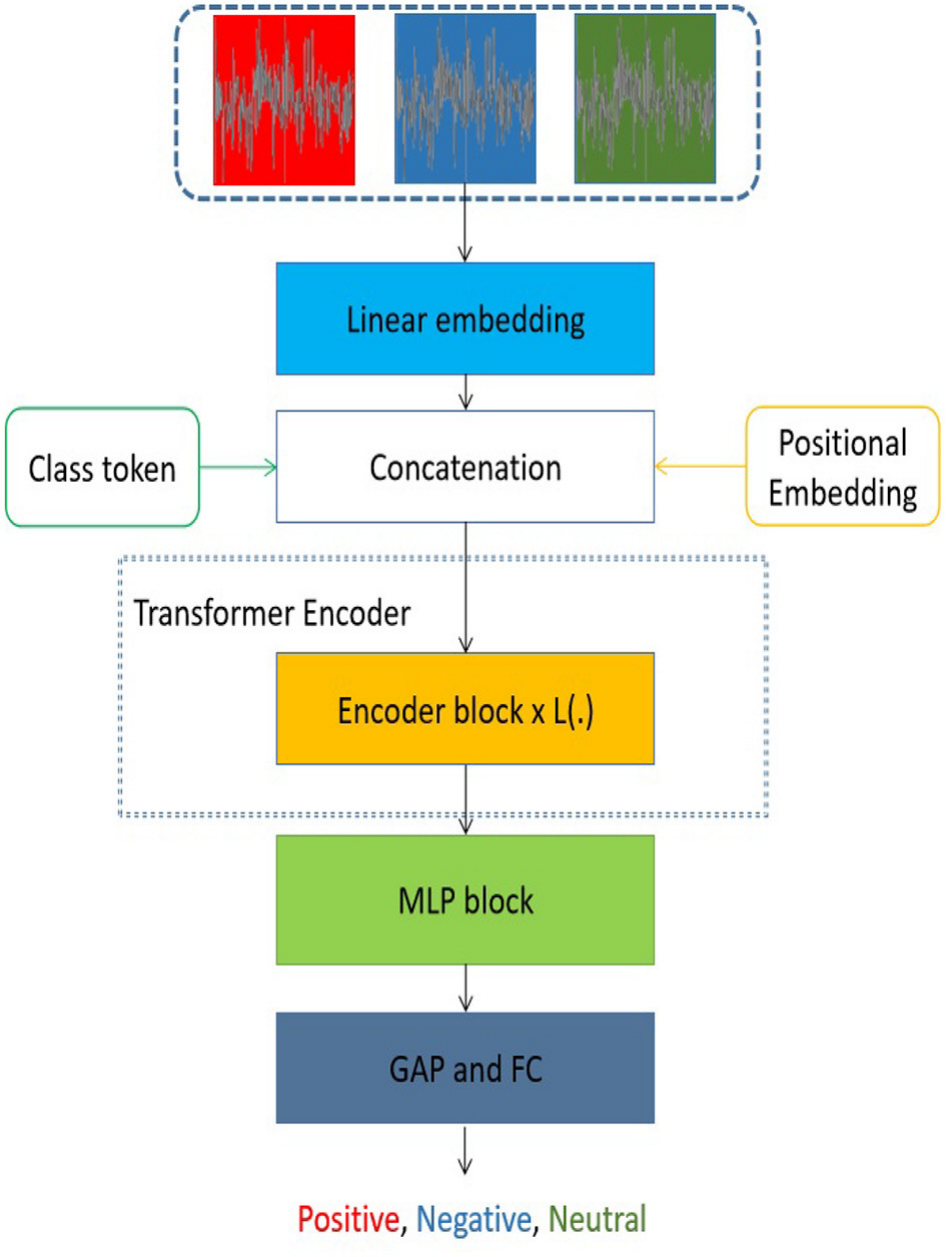
The architectural design of the proposed transformer model. The symbol L is used to describe the number of encoder blocks in the proposed model. The proposed model has several variants, with L(.) being either 12, 18, or 24. The abbreviation GAP is used to denote global average pooling, while FC represents fully-connected.

The transformer model presented in Figure 2 draws inspiration from the architecture of the vision transformer (Dosovitskiy et al., 2020). The suggested transformer model comprises three main components: (1) a linear embedding layer, (2) an encoder block, and (3) a multiple-layer perception (MLP) block. Initially, the linear embedding unit was utilized to turn a sequence of EEG data into a fixed-length input for the suggested transformer model. The flattened embedding includes the class token of the music emotion for each series of EEG data. In addition, the linear embedding is constructed by including the positional embedding, which encodes the sequential order of an individual EEG signal inside a sequence of EEG signals. It should be noted that every input sequence of EEG data pertains to the identical category of emotion elicited by music. Furthermore, the pivotal self-attention module (Fan et al., 2021; Liu et al., 2021; Wang et al., 2021), which aims to reveal the connections among distant EEG data, is located within the encoder block. In order to create a cohesive encoder module, it is necessary for the encoder block to be iteratively repeated. In addition to the self-attention layer included in each encoder block, there are many additional sorts of layers, namely layer normalization, dropout, and MLP block. The generation of representations for music emotion EEG signals may be achieved by the utilization of stacked transformer encoder blocks. Ultimately, the use of the MLP block was implemented to get the classification result by integrating a global average pooling (GAP) layer and a fully connected (FC) layer, commonly referred to as a linear layer. The transformer model under consideration has the potential to significantly expand the scope of receptive fields in comparison to designs based on CNNs. Additionally, the recovered representation from the multi-channel EEG data encompasses both local information pertaining to a series of signals and the global association between signals that are far apart.

**Figure 3:**
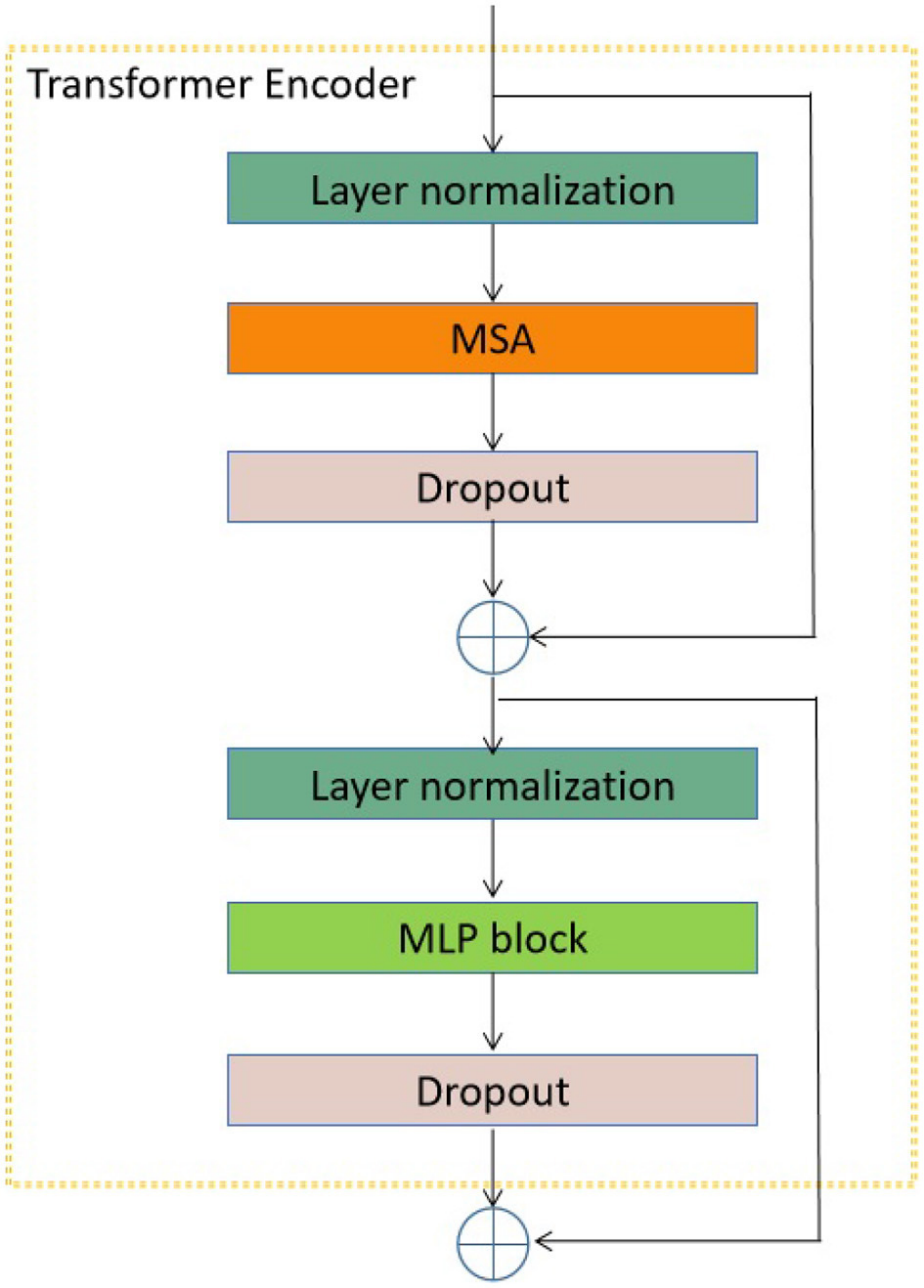
The encoder block utilized in the transformer model under consideration. The acronym MSA refers to multi-head self attention.

In the proposed transformer model, the input sequences consist of individual EEG signals, each spanning a duration of 1 second and including 30 channels. Subsequently, the EEG signal sequence was flattened and transformed into a vector. In addition, it should be noted that the encoder block is iterated a varying number of times (12, 18, or 24) across different versions of the proposed transformer model. Furthermore, the structural composition of this encoder block is illustrated in Figure 3.

**Figure 4:**
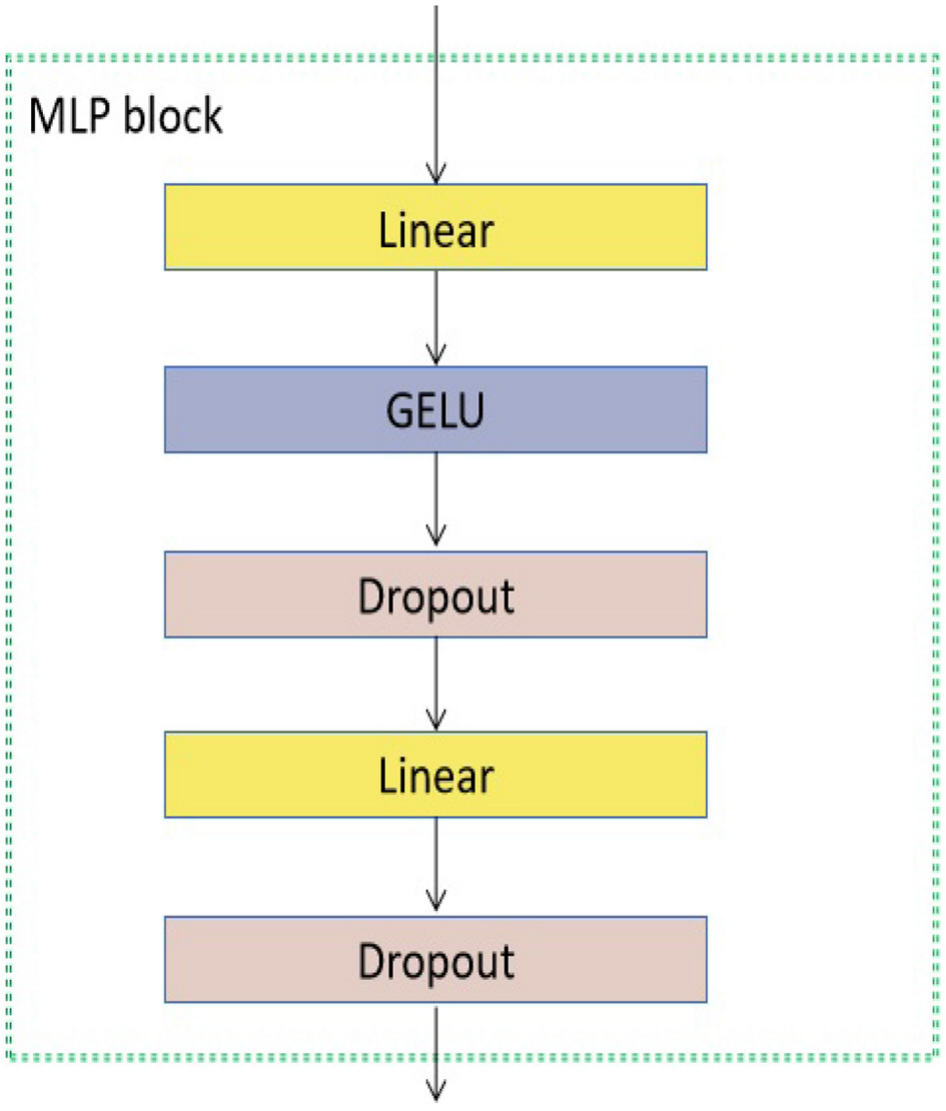
The MLP block used in the proposed transformer model. GELU denotes the activation function (Lee, 2023).

As seen in Figure 3, the encoder block has many components, namely layer normalization, MSA, dropout, and MLP block. The study did not include a comprehensive examination of the MSA unit due to its extensive coverage in existing studies (Vaswani et al., 2017; Dosovitskiy et al., 2020). The unit consisting of *H* heads was employed to assess the similarity between a query and its associated keys based on the assigned weight for each value (Vaswani et al., 2017). Furthermore, the utilization of the Layer normalizing module is employed to calculate the mean and variance required for normalizing from the entirety of the inputs to the neurons within a layer throughout a singular training instance (Ba et al., 2016). In this study, the dropout layer (Choe and Shim, 2019) is utilized as a regularization technique to mitigate the risk of overfitting. The architecture of the MLP block is seen in Figure 4.

The proposed technique allows for the formulation of the process of music emotion categorization in Equation 1–4:


(1) $$\begin{array}{l} z_{0}=\left[x_{class};x_{p}^{1}E;x_{p}^{2}E;...;x_{p}^{N}\right]+E_{position}, \end{array}$$

where the variable *z*_0_ represents the output of the linear embedding layer. In this context, *N* = 30 represents the number of channels used as input. The variables *x*_*class*_ and *E*_*position*_ refer to the class token and positional embedding, respectively.


(2) $$\begin{array}{l} z_{l}^{\prime }=MSA\left(LN\left(z_{l-1}\right)\right)+z_{l-1}, \end{array}$$

(3) $$\begin{array}{l} z_{l}=MLP\left(LN\left(z_{l}^{\prime }\right)\right)+z_{l}^{\prime }, \end{array}$$

(4) $$\begin{array}{l} y=LN\left(z_{L}^{0}\right), \end{array}$$

where the layer normalization unit is denoted as *LN*(.), where *z*_*l*_ represents the output of layer *l*, and *y* represents the output classification outcome.

## 3 Experimental results

### 3.1 Implementation details

The transformer model described in this study was constructed using the PyTorch framework (Paszke et al., 2019). The computational resources employed for the implementation were four NVidia RTX 3080 Graphical Processing Units (GPUs) with a total of 64 GB RAM. The best parameters of the proposed network were discovered using a trial and error technique. The learning rate is configured to be 0.004, accompanied by a weight decay of 0.05. Subsequently, a 10-fold cross-validation procedure was employed to assess the resilience of the suggested methodology. Initially, the input EEG data were partitioned into ten equitably sized groups. During each iteration, one out of the 10 groups was designated as the testing set, while the remaining nine groups were utilized as the training set. Hence, the mean result of 10 iterations was utilized as the ultimate output.

Furthermore, the assessment measures utilized in the experiments involved sensitivity, specificity, and accuracy. The mathematical formulation of these metrics is elucidated in in Equations 5–7.


(5) $$\begin{array}{l} Sensitivity=\frac{TP}{TP+FN}, \end{array}$$

(6) $$\begin{array}{l} Specificity=\frac{TN}{TN+FP}, \end{array}$$

(7) $$\begin{array}{l} Accuracy=\frac{TP+TN}{TP+FN+TN+FP}, \end{array}$$

where TP, FN, TN, and FP represent the terms true positive, false negative, true negative, and false positive, respectively.

### 3.2 Outcome of the proposed approach

Table 2 presents a summary of the average values and standard deviations (SD) obtained from the proposed method in the binary classification task, specifically in terms of average accuracy, sensitivity, and specificity. The average accuracy was found to be 96.85%, while the sensitivity and specificity were measured at 95.17% and 95.69% respectively. Furthermore, in the ternary categorization, the outcome rates were recorded as 95.74%, 94.32%, and 95.25%.

**Table 2:** The proposed transformer model exhibits binary and ternary classification outcomes (average values and standard deviations).

**Number of classes**	**Accuracy (%)**	**Sensitivity (%)**	**Specificity (%)**
Binary	96.85 (1.73)	95.17 (1.68)	95.69 (2.01)
Ternary	95.74 (2.32)	94.32 (1.97)	95.25 (1.69)

**Figure 5:**
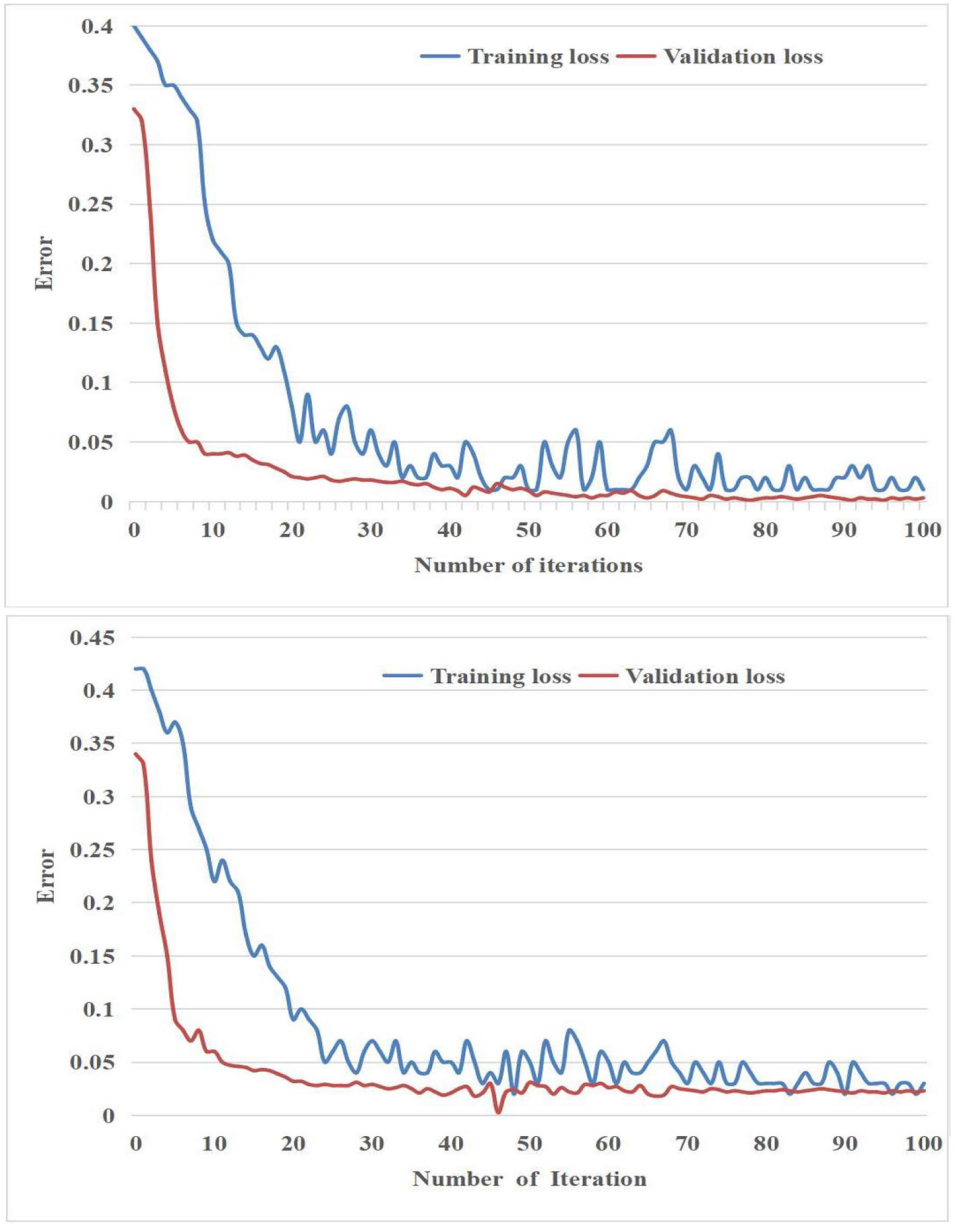
The suggested model's inaccuracy in **(Top)** binary classification and **(Bottom)** ternary classification.

Furthermore, the loss curves of the suggested methodology throughout both the training and validation procedures were illustrated in Figure 5 It should be noted that the results presented in Figure 5 only include the initial 100 iterations of both the training and validation processes.

### 3.3 Comparison experiments between the state-of-the-arts and the proposed approach

To assess the efficacy of our suggested technique for music-evoked emotion categorization, we conducted comparative tests between our work and the state-of-the-art algorithms. Tables 2–4 present a comparative analysis of the current state-of-the-art deep learning models and our proposed approach. The proposed methodology demonstrated superior performance compared to the current leading method. *To note that we did not take the traditional machine learning models (Qiu et al., 2022) into the comparison since they usually relied on manually-designed features*. The comparison experiments included the following models: U-Net (Ronneberger et al., 2015), Mask R-CNN (He et al., 2017), ExtremeNet (Zhou et al., 2019), TensorMask (Chen et al., 2019), 4D-CRNN (Shen et al., 2020), FBCCNN (Pan and Zheng, 2021), MTCNN (Rudakov, 2021), SSGMC (Kan et al., 2022) for the CNN-based models, and MViT (Fan et al., 2021), PVT (Wang et al., 2021), PiT (Heo et al., 2021), Swin Transformer (Liu et al., 2021), and GPViT (Yang et al., 2022) for the transformer-based models.

**Table 3:** Binary classification comparison between the state-of-the-arts and ours.

**Method**	**Accuracy (%)**	**Sensitivity (%)**	**Specificity (%)**
U-Net (Ronneberger et al., 2015)	88.56	88.71	89.05
Mask R-CNN (He et al., 2017)	87.43	86.39	86.56
ExtremeNet (Zhou et al., 2019)	89.49	89.87	88.51
TensorMask (Chen et al., 2019)	90.56	90.18	91.27
4D-CRNN (Shen et al., 2020)	92.57	92.32	93.08
FBCCNN (Pan and Zheng, 2021)	92.53	91.68	91.24
MTCNN (Rudakov, 2021)	93.02	93.55	94.17
SSGMC (Kan et al., 2022)	94.82	94.18	94.23
MViT (Fan et al., 2021)	90.42	91.39	90.72
PVT (Wang et al., 2021)	92.27	91.15	92.01
PiT (Heo et al., 2021)	93.53	92.85	93.78
Swin Transformer (Liu et al., 2021)	95.32	94.64	94.37
GPViT (Yang et al., 2022)	96.38	94.88	95.27
The proposed approach	96.85	95.17	95.69

**Table 4:** Ternary classification comparison between the state-of-the-arts and ours.

**Method**	**Accuracy (%)**	**Sensitivity (%)**	**Specificity (%)**
U-Net (Ronneberger et al., 2015)	85.52	83.86	84.20
Mask R-CNN (He et al., 2017)	85.24	84.21	85.41
ExtremeNet (Zhou et al., 2019)	86.28	83.17	84.53
TensorMask (Chen et al., 2019)	88.32	86.51	87.02
4D-CRNN (Shen et al., 2020)	91.57	92.24	91.89
FBCCNN (Pan and Zheng, 2021)	91.27	91.38	92.24
MTCNN (Rudakov, 2021)	92.21	92.19	93.43
SSGMC (Kan et al., 2022)	92.18	91.57	94.28
MViT (Fan et al., 2021)	92.15	91.93	92.78
PVT (Wang et al., 2021)	91.23	90.46	91.37
PiT (Heo et al., 2021)	92.43	92.14	91.62
Swin transformer (Liu et al., 2021)	92.57	91.38	93.27
GPViT (Yang et al., 2022)	93.14	92.25	93.18
The proposed approach	95.74	94.32	95.25

In order to conduct a comprehensive evaluation of the proposed approach, we proceeded to assess its performance alongside several state-of-the-art algorithms (Shawky et al., 2018; Yang et al., 2018; Xing et al., 2019; Yang et al., 2019; Shen et al., 2020; Pan and Zheng, 2021; Rudakov, 2021; Kan et al., 2022; Zhang et al., 2022) using the publicly accessible DEAP dataset (Koelstra et al., 2011). The results of this evaluation are presented in Table 5.

**Table 5:** Comparison between the state-of-the-arts and ours on DEAP dataset (Koelstra et al., 2011).

**Method**	**Detail**	**Accuracy**	
		**Valence**	**Arousal**
3DCNN (Shawky et al., 2018)	CNN	88.52	89.36
CNN-LSTM (Yang et al., 2018)	LSTM	92.43	89.51
SAE-LSTM (Xing et al., 2019)	LSTM	86.32	81.27
Multi-column CNN (Yang et al., 2019)	CNN	93.81	94.15
4D-CRNN (Shen et al., 2020)	CRNN	95.34	93.62
FGCCNN (Pan and Zheng, 2021)	CNN	91.72	90.28
MTCNN (Rudakov, 2021)	CNN	95.34	95.49
GANSER (Zhang et al., 2022)	GAN	94.18	93.58
SSGMC (Kan et al., 2022)	Contrastive learning	96.12	94.62
The proposed approach	Transformer	97.41	97.02

## 4 Discussion

Based on the empirical findings, it can be concluded that this approach exhibits greater efficacy compared to the existing state-of-the-art algorithms. It is worth mentioning that the comparative trials encompassed both CNN-based and transformer-based models. In contrast to CNN-based models, the suggested model has the capability to extract global connections between long-range multi-channels in EEG data, in addition to the local information already present in the EEG signals. In contrast to transformer-based models (He et al., 2017; Chen et al., 2019; Zhou et al., 2019; Wu et al., 2020; Fan et al., 2021; Heo et al., 2021; Wang et al., 2021), the proposed approach has been specifically optimized to accommodate the unique characteristics of multi-channel EEG signals. For instance, the linear embedding layer of the proposed approach has been tailored to effectively align with the structural properties of multi-channel EEG signals. Furthermore, the outcomes shown in the ablation research also exhibited the efficacy of self-attention modules and encoder blocks.

### 4.1 Ablation study

As demonstrated in Table 6, the optimal configuration of the primary hyper-parameters was determined through comparison experiments. These experiments involved testing different combinations of the number of heads (*H*) in the MSA module and the number of transformer encoder layers (*L*) on a dataset that was manually collected and constituted 50% of the total dataset. The trials solely included binary music emotion categorization in order to streamline the ablation study procedure.

**Table 6:** The impact of *H* and *L* on the performance of the proposed model in binary classification.

**Model**	**Number of heads (H)**	**Number of layers (L)**	**Accuracy (%)**
M_4_4	4	4	90.08
M_4_8	4	8	90.37
M_8_4	8	4	91.15
M_8_8	8	8	91.63
M_8_12	8	12	93.35
M_12_12	12	12	93.21
M_16_12	16	12	94.16
M_8_18	8	18	94.58
M_12_18	12	18	95.39
M_16_18	16	18	95.65
M_8_24	8	24	96.28
M_12_24	12	24	96.12
M_16_24	16	24	**96.53**

The bold value represents the best performance of accuracy with 16 heads and 24 layers.

Therefore, the suggested model exhibits an ideal configuration while utilizing 16 heads (*H* = 16) and 24 layers (*L* = 24).

### 4.2 Limitations and future research

In addition, this study possesses certain limitations in addition to its contributions. The tests solely focused on the binary and ternary classification problems. In order to enhance the evaluation of the proposed approach, it is recommended to integrate the categorization of other types of emotions and employ a multi-label classification methodology. Meanwhile, this study adopted an offline learning strategy since the vision transformer-based models suffering from high resource occupancy. In addition, this study did not take cross-subject emotion recognition (He et al., 2021; Pan et al., 2023) into consideration, which may affect the applicability and universality of this study.

In subsequent investigations, further electroencephalography (EEG) data pertaining to the elicitation of emotions through music will be gathered. Furthermore, the suggested methodology holds potential for the identification of emotions across a wide range of applications.

## 5 Conclusion

The present work introduces a transformer model as a means of classifying music-evoked emotions. The model under consideration consists of three distinct phases, namely linear embedding, transformer encoder, and MLP layer. The purpose of the first phase is to generate flattened input features for the proposed model. These features are aimed to extract both local and global correlations between the multi-channel EEG data. Additionally, the MLP blocks aim to enhance the classification outcome. This study presents an initial implementation of a vision transformer-based model for the purpose of music emotion identification.

## Data availability statement

The raw data supporting the conclusions of this article will be made available by the authors, without undue reservation.

## Ethics statement

The studies involving humans were approved by the Shandong Management University's Human Research Ethics Committee. The studies were conducted in accordance with the local legislation and institutional requirements. The participants provided their written informed consent to participate in this study.

## Author contributions

DW: Writing – review & editing, Formal analysis, Validation. JL: Writing – original draft, Supervision, Project administration, Methodology, Funding acquisition, Formal analysis, Data curation, Conceptualization. HC: Writing – original draft, Validation, Investigation, Formal analysis, Data curation. YZ: Writing – original draft, Validation, Investigation, Data curation.
