# Corrigendum: Music-evoked emotions classification using vision transformer in EEG signals

**DOI:** 10.3389/fpsyg.2024.1422498

**Published:** 2024-05-23

**Authors:** Dong Wang, Jian Lian, Hebin Cheng, Yanan Zhou

**Affiliations:** ^1^School of Intelligence Engineering, Shandong Management University, Jinan, China; ^2^School of Arts, Beijing Foreign Studies University, Beijing, China

**Keywords:** music-evoked emotion, emotion classification, electroencephalographic, deep learning, transformer

In the published article, there was an error. It was not clearly denoted that authors Dong Wang and Jian Lian contributed equally to this work. The correct information is shown below.

Dong Wang^1†^, Jian Lian^1†^, Hebin Cheng^1*^ and Yanan Zhou^2*^

^†^These authors have contributed equally to this work

The authors apologize for this error and state that this does not change the scientific conclusions of the article in any way. The original article has been updated.

