# Corrigendum: Music-evoked emotions classification using vision transformer in EEG signals

**DOI:** 10.3389/fpsyg.2024.1466709

**Published:** 2024-08-12

**Authors:** Dong Wang, Jian Lian, Hebin Cheng, Yanan Zhou

**Affiliations:** ^1^School of Information Science and Electrical Engineering, Shandong Jiaotong University, Jinan, China; ^2^School of Intelligence Engineering, Shandong Management University, Jinan, China; ^3^School of Arts, Beijing Foreign Studies University, Beijing, China

**Keywords:** music-evoked emotion, emotion classification, electroencephalographic, deep learning, transformer

In the published article, there were errors in the affiliations for author Dong Wang. As well as ‘School of Intelligence Engineering, Shandong Management University, Jinan, China', this author should also be affiliated to “School of Information Science and Electrical Engineering, Shandong Jiaotong University, Jinan, China”. The corrected author affiliations are shown below.

Dong Wang^1, 2†^, Jian Lian^2†^, Hebin Cheng^2*^ and Yanan Zhou^3*^

^1^School of Information Science and Electrical Engineering, Shandong Jiaotong University, Jinan, China

^2^School of Intelligence Engineering, Shandong Management University, Jinan, China

^3^School of Arts, Beijing Foreign Studies University, Beijing, China

The authors apologize for this error and state that this does not change the scientific conclusions of the article in any way. The original article has been updated.

